# The Illinois Anatomical Law

**Published:** 1885-08

**Authors:** 


### News Items.

The Illinois Anatomical Law.

An Act to promote the science of medicine and surgery in the State of Illinois. Approved June 26, 1885. In force July 1, 1885.

Section 1. *Be it enacted by the People of the State of Illinois, represented in the General Assembly:* That superintendents of penitentiaries, houses of correction and bridewells, wardens of hospitals, insane asylums and poor houses, coroners, sheriffs, jailers, city and county undertakers, and all other State, county, town and city officers, in whose custody the body of any deceased person, required to be buried at public expense, shall be, shall give permission to any physician or surgeon, (a licentiate of the State board of health), or any medical college or school, public or private, of any city, town or county, upon his or their request therefor to receive and remove free of charge or expense, after having given proper notice to relatives or guardians of the deceased, the bodies of such deceased persons to be buried at public expense, to be by him or them used within the State, for advancement of medical science; preference being given to medical colleges or schools, public or private; said bodies being distributed to and among same, equitably; the number assigned to each, being in proportion to the students of each college or school: *Provided however,* that if any person claiming to be, and satisfying the proper authorities that he is of kindred to the deceased, shall ask to have the body for burial, it shall be surrendered for interment: *And*

*provided further*, that any medical college or school, public or private, or any officers of the same, that shall receive the bodies of deceased persons for the purpose of scientific study, under the provisions of this act, shall furnish the same to students of medicine and surgery, who may be under their instruction, at a price not exceeding the sum of five dollars for each and every such deceased body so furnished.

SEC. 2. Any physician or surgeon, (a licentiate of the Illinois State Board of Health) or any medical college or school, public or private, before receiving any dead body or bodies, shall give to the proper authority, surrendering the same to him or them, a sufficient bond that said body or bodies shall be used only for the promotion of medical science within this State; and whoever shall use said body or bodies for any other purpose, or shall remove the same beyond the limits of this State; and whosoever shall sell or buy any such body or bodies or shall traffic in the same, shall be deemed guilty of a misdemeanor, and shall, on conviction, be fined in a sum of not less than one hundred dollars, and be imprisoned in the county jail for a term not less than thirty days nor more than one year; the fine accruing from such conviction, to be paid into the school fund of the county where the offense shall have been committed.

SEC. 3. Any officer refusing to deliver the remains or body of any deceased person when demanded in accordance with the provisions of this act, shall pay a penalty of not less than fifty dollars, nor more than one hundred dollars for the first offense, and for the second offense, a penalty of not less than one hundred dollars, nor more than five hundred dollars; and for a third offense, or any offense thereafter, the penalty of not less than five hundred dollars, or to be imprisoned in the county jail not less than six nor more than twelve months, or both, at the discretion of the court; such penalties to be sued for by the health department, as the case may be.

SEC. 4. It shall be the duty of preceptors, professors and teachers, and all officers of medical colleges and schools, public or private, who shall receive any dead body or bodies, in pursuance of the provisions of this act, decently to bury, in some public cemetery, or to cremate the same in a furnace properly constructed for that purpose, the remains of all bodies, after they shall have answered the purpose of study aforesaid, and for any neglect or violations of the provisions of this act, the party or parties so neglecting, shall on conviction, forfeit or pay a penalty of not less than fifty dollars, nor more than one hundred dollars, or be imprisoned in the county jail not less than six nor more than twelve months, or both, at the discretion of the court; such penalties to be sued for by school officers, or any person interested therein, for the benefit of the school fund of the county in which the offense shall have been committed.

SEC. 5. An act entitled "An act to promote the science of medicine and surgery in the State of Illinois," approved February 16, 1874, in force July 1, 1874, is hereby repealed.

